# αA-crystallin R49C^neo ^mutation influences the architecture of lens fiber cell membranes and causes posterior and nuclear cataracts in mice

**DOI:** 10.1186/1471-2415-9-4

**Published:** 2009-07-20

**Authors:** Usha P Andley

**Affiliations:** 1 Department of Ophthalmology and Visual Sciences, Washington University School of Medicine, St Louis, Missouri, USA

## Abstract

**Background:**

αA-crystallin (CRYAA/HSPB4), a major component of all vertebrate eye lenses, is a small heat shock protein responsible for maintaining lens transparency. The R49C mutation in the αA-crystallin protein is linked with non-syndromic, hereditary human cataracts in a four-generation Caucasian family.

**Methods:**

This study describes a mouse cataract model generated by insertion of a neomycin-resistant (neo^r^) gene into an intron of the gene encoding mutant R49C αA-crystallin. Mice carrying the neo^r ^gene and wild-type *Cryaa *were also generated as controls. Heterozygous knock-in mice containing one wild type gene and one mutated gene for αA-crystallin (WT/R49C^neo^) and homozygous knock-in mice containing two mutated genes (R49C^neo^/R49C^neo^) were compared.

**Results:**

By 3 weeks, WT/R49C^neo ^mice exhibited large vacuoles in the cortical region 100 μm from the lens surface, and by 3 months posterior and nuclear cataracts had developed. WT/R49C^neo ^mice demonstrated severe posterior cataracts at 9 months of age, with considerable posterior nuclear migration evident in histological sections. R49C^neo^/R49C^neo ^mice demonstrated nearly complete lens opacities by 5 months of age. In contrast, R49C mice in which the neo^r ^gene was deleted by breeding with CreEIIa mice developed lens abnormalities at birth, suggesting that the neo^r ^gene may suppress expression of mutant R49C αA-crystallin protein.

**Conclusion:**

It is apparent that modification of membrane and cell-cell interactions occurs in the presence of the αA-crystallin mutation and rapidly leads to lens cell pathology *in vivo*.

## Background

Cataracts involving mutations in lens crystallin genes have received considerable attention in recent years [1-5]. Vertebrate lens crystallins are divided into two families, α and βγ. α-crystallin is essential for lens transparency and accounts for nearly 50% of the protein mass in human lenses. It is a large multimeric complex with an aggregate molecular mass of 500,000–1,200,000 Da, and is isolated from lens fiber cells as a complex of αA- and αB-crystallin in a 3:1 stoichiometry [6]. αA-crystallin/HSPB4 is a member of the small heat shock protein family, which also includes αB-crystallin/HSPB5 and Hsp27/HSPB1 [7]. The etiology of lens disease is diverse, but a common pathological endpoint is the formation of large protein aggregates that scatter light. The capacity of α-crystallins to efficiently trap aggregation-prone denatured proteins is thought to delay age-related cataract in humans. α-crystallin and homoaggregates of αA and αB-crystallin interact *in vitro *with non-native proteins and prevent their irreversible aggregation and insolubilization [8]. Using recombinant proteins, αA and αB-crystallin have been shown to interact with many partially denatured substrates. Their stability, dynamic properties, and ability to transition from large assemblies to smaller dimeric and monomeric species appear to be critical for this chaperone ability [9-13]. The binding of αA and αB-crystallin to misfolded proteins occurs with a high efficiency [14]; however, once all the α-crystallin in lens fiber cells has been depleted, the concentration of irreversibly denatured proteins could increase, resulting in cataract. αA-crystallin binds to lens cell membranes and this association is increased in cataracts. Indeed, many models of cataract involve alterations in lens cell membranes [15-17]. The interaction of α-crystallin with the cytoskeleton is also of major importance in maintaining lens transparency [18-22].

αA-crystallin transcripts are present in mouse lens epithelium at embryonic day 10.5 and continue to be expressed during embryonic development and postnatally [23]. Studies on α-crystallin knockout mice suggest that loss of αA-crystallin may increase the concentration of unstable proteins and affect the solubility of αB-crystallin and γ-crystallin [24,25]. In addition to its essential role in the optical and refractive properties of the eye lens, αA-crystallin performs other functions; αA-crystallin knockout mice exhibit increased lens epithelial cell death and reduced cell proliferation [26,27], and αA-crystallin is also expressed in the retina, brain, spleen, and thymus [28] although its role in these tissues is not fully understood.

Transfection studies show that α-crystallins protect cells from stress-induced apoptosis [27]. Both αA and αB-crystallin are negative regulators of apoptosis in lens cells [29,30], and αA-crystallin knockout lens epithelial cells have a higher level of cell death than wild type cells [31]. Thus, cellular protection by crystallins may delay the onset of age-related and hereditary cataracts.

Several single-point mutations in α-crystallins have been linked with hereditary human cataracts [1,32-37]. In αA-crystallin, R49C and W9X mutations in the N-terminal domain, and R116C, R116H, and G98R mutations in the C-terminal domain cause human cataracts, as do C-terminal R120G, 450delA, D140N mutations in αB-crystallin. Many of these mutations are inherited by autosomal dominant mechanisms. Study of these αA-crystallin gene mutations would enhance our understanding of the mechanisms of cataract formation [1,5,32,34,37-39]. Mutations in αA-crystallin increase the abundance of aggregation-prone proteins. *In vitro *studies suggest that mutant αA-crystallin may aggregate causing increased co-precipitation of substrate proteins [40-43]. The most common effect of single-point mutations and truncations is an increase in the size of the oligomeric complex [44-46]. The R49C mutant of αA-crystallin has a slightly higher mass and radius of gyration, but the main effect of the mutation is increased protein insolubility [42]. Arginine is a highly charged residue, and its replacement by the less polar cysteine might alter protein assembly and solubility. Moreover, the positive charge on arginine 49 has been highly conserved during evolution, and is important for *in vitro *chaperone activity of αA-crystallin [47,48].

Several model systems are available to study the effect of single-point mutations of crystallin genes. Transgenic mice have been used to investigate the effect of the R116C mutation in αA-crystallin *in vivo *[49], and several studies have used transfected cells expressing mutant proteins [1,38,50]. Naturally occurring and mutagenesis-induced mouse models have also been analyzed [51-54]. Naturally occurring αA-crystallin mutations in mice are *Cryaa lop1 *(R54H), and *Cryaa *R54C mutations in the N-terminal region, and Y118D and *Cryaa Aey7 *(V124E) mutations in the C-terminal domain. While significant information has been obtained by studying mutant αA-crystallin *in vitro*, such studies have certain limitations. Mutations affecting protein interactions at low concentrations may have little relevance to how they associate *in vivo *at higher concentrations in the lens. Thus, an optimal model must investigate the effect of the mutation *in vivo*. To study the mechanism of hereditary cataracts, this laboratory recently generated R49C knock-in mice and compared wild type lenses with heterozygous and homozygous mutant lenses to investigate gene dosage effects [42,55]. While generating the R49C knock-in mice, in which the floxed neo cassette was deleted by mating with Cre deletor mice, we serendipitously created WT/R49C^neo ^and R49C^neo^/R49C^neo ^mouse cataract models which show milder lens effects *in vivo*, thus allowing examination of the effects of the mutant protein at potentially changed levels of expression. The aim of the present study was to characterize the lens abnormalities in WT/R49C^neo ^and R49C^neo^/R49C^neo ^mice and compare these with previous reports of WT/R49C and R49C/R49C lenses [42,55].

## Methods

### Animals

Mice were maintained at Washington University Division of Comparative Medicine by trained veterinary staff. All protocols and animal procedures were approved by the Washington University Animal Studies Committee. Lens opacity was monitored by slit lamp biomicroscopy. The left eye of the animals was examined, and pupils were dilated with a mixture of 10% phenylephrine hydrochloride and 1% tropicamide (Alcon, Fort Worth, Texas). Mice carrying the R49C^neo ^locus were generated from one embryonic stem cell clone (129 background) as previously described [55]. This locus carries a neomycin cassette flanked by lox P sites inserted into an *XhoI *site in intron 1 of *Cryaa*. One ES clone with the neo insertion but no *Cryaa *mutation was used to generate WT/WT^neo ^and WT^neo^/WT^neo ^mice. Two lines of mice expressing the mutation in *Cryaa*, R49C^neo^KI3 and R49C^neo^KI4, were bred. Another mouse line, WT^neo^KI2, expressing wild type *Cryaa *was also generated on 129 background. Mice containing one copy of the targeted knock-in allele (heterozygous mice) were interbred to generate homozygous mice. Genotyping primers were used to identify the knock-in construct containing the neomycin cassette as described previously [55]. Transgenic mice expressing Cre-EIIa on a C57BL/6 background were bred with homozygous *Cryaa *knock-in mice to delete the neo^r ^gene. These mice were described in previous studies [42,55].

### Slit lamp examination

Dilated mouse eyes were examined in unanesthetized mice. Stages of cataracts were defined as follows: Stage 0 – clear lens; Stage 1 – loss of normal appearance of posterior lens and prominence of y-suture line; Stage 2 – discrete posterior changes accompanied by light nuclear opacity; Stage 3 – nearly mature cataract, involving approximately three-fourths of the lens with bubbles and opacity; Stage 4 – completely mature cataract involving the cortex with bubbles and vacuoles.

### Histology

For conventional histology, eyes were fixed overnight at 4°C in formalin (Sigma, St. Louis, MO). After a thorough wash in phosphate-buffered saline, lenses were dehydrated through graded acetone and infiltrated in methacrylate resin (H-8100; Technovit, Kulzer, Germany) according to the following schedule: 1:2 resin:acetone, 1 day; 1:1 resin:acetone, 1 day; 100% resin, 4 days. Blocks were polymerized for 1 hour at 4°C. Sections (3 μm) were cut and stained with haematoxylin and eosin.

### Immunofluorescence

Immunofluorescence was performed as previously described [31]. Primary antibody to lens membrane intrinsic protein MIP was purchased from Alpha Diagnostics International. Alexa^568^-labeled secondary antibody (Molecular Probes) was used at 1:500 dilution.

### Immunoblotting

Lens extracts were separated into water-soluble and insoluble fractions, and examined by SDS-PAGE and immunoblotting with an antibody to α-crystallin [56]. Protein concentration of the water-soluble fractions was determined by the bicinchoninic acid (BCA) protein assay according to the manufacturer's instructions (Thermo Scientific-Pierce Chemical Co, Rockford IL). 40–50 μg of extract was loaded on the gel. The primary antibody was either a polyclonal antibody (used at 1:500 dilution) to total α-crystallin or a monoclonal antibody (a gift from Dr. Paul FitzGerald; used at 1:100 dilution) to αA-crystallin, and horseradish peroxidase-conjugated secondary antibodies as described previously [27,42,56]. The relative intensity of the mouse αA-crystallin band at approximately 20 kDa was analyzed by Image J software. The decrease in αA-crystallin soluble protein with increasing numbers of R49Cneo^r^-containing genes was calculated as the mean of three independent experiments.

### Cryoimmuno electron microscopic analysis

Lens sections were examined by cryoimmuno electron microscopy. Sections (50–80 nm) were treated with a primary monoclonal antibody to αA-crystallin [27] and anti-mouse IgG conjugated with 18 nm gold particles as secondary antibody (Sigma). Specimens were stained with uranyl acetate and examined in a 1200EX transmission electron microscope as described previously [57].

### Analytical chromatography and analysis

WT/WT^neo ^and WT/R49C^neo ^mouse lenses (1.5 to 2.5 month old) were used to isolate water-soluble proteins [42]. Six lenses of each genotype were homogenized in 500 μl of phosphate-buffered saline (Sigma) and the water-soluble protein fraction was separated by centrifugation at 15,000 g. FPLC chromatography was performed on a chromatography system containing an inline detector for UV absorption at 280 nm. A 16 cm × 60 cm Superdex 200 column (GE Healthcare Biosciences) equilibrated with 10 mM Tris.Cl; pH 6.8 containing 100 mM NaCl, 1 mM DTT and 0.5 mM EDTA was used. Proteins were injected at 1 mg/ml for different chromatographic runs. One ml fractions were collected, and proteins mass was determined by calibrated molecular mass standards. The area under each peak was used to determine changes in abundance of α, β and γ-crystallin fractions. The results are representative of 6 lenses per genotype. The analysis was repeated with almost the same results.

## Results

**Figure 1:**
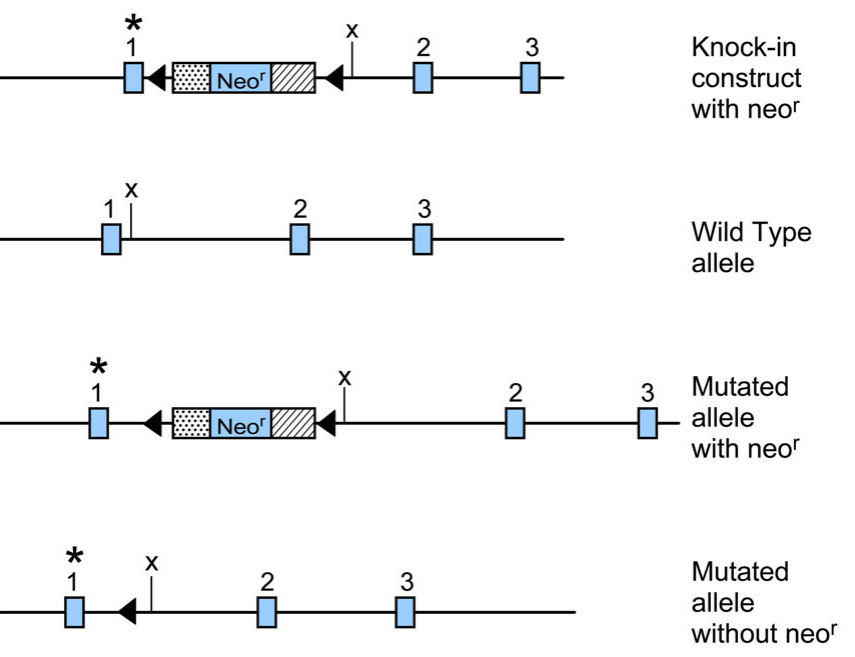
**Plasmid construct used to generate R49C^neo ^gene knock-in mice**. The 5' and 3' arms of the αA-crystallin gene (*Cryaa*) were cloned into a vector containing the floxed neomycin (neo) cDNA. Mutagenesis was performed to mutate amino acid arginine 49 of αA-crystallin to cysteine (R49C). The asterisk above exon 1 indicates the mutation. The numbered blue rectangles indicate exons. The filled triangles are loxP sites and X denotes the *XhoI *site. Mouse embryonic stem (ES) cells SCC-10 were electroporated with the mutant plasmid, and clones testing positive for neo were identified and used to generate R49C^neo ^αA-crystallin knock-in mice (WT/R49C^neo^). One clone containing wild type (WT) αA-crystallin cDNA and neo was also analyzed and used to generate mice with the neo allele but no mutation (WT/WT^neo^).

**Figure 2:**
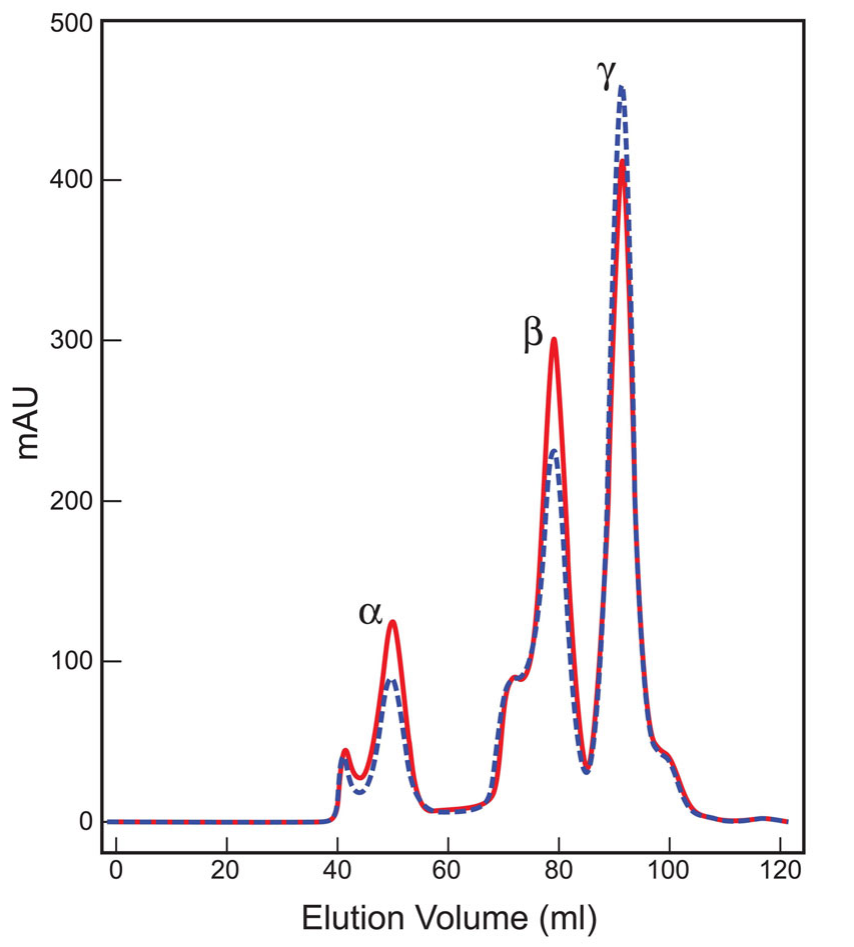
**FPLC analysis of crystallins in WT/WT^neo ^and WT/R49C^neo ^mouse lenses**. UV absorption profile of water-soluble mouse lens proteins separated from WT/WT^neo ^*(red) *and WT/R49C^neo ^*(blue) *by gel permeation chromatography. Proteins were pooled from six lenses of each genotype at ~2-month-old. mAU represents milli absorbance units at 280 nm.

While constructing a gene knock-in mouse model of R49C αA-crystallin, we generated mice carrying a floxed neo^r ^gene in an intron of the *Cryaa *gene (Figure 1). Mating these to a mouse expressing Cre recombinase resulted in removal of the neo cassette, leaving the loxP site adjacent to exon 1 of the *Cryaa *gene, and producing a functional but less active mutant allele [42,55]. Two lines of R49C^neo ^mice, R49C^neo^KI 3 and R49C^neo^KI 4, were generated from a single ES cell clone, and the results obtained from these two lines were very similar. The knock-in mice were viable and bred normally. Heterozygous WT/R49C^neo ^mice were intercrossed to produce R49C^neo^/R49C^neo ^homozygous offspring. A control mouse line was also generated from an ES cell clone that had the neomycin cassette inserted, but lacked the R49C αA-crystallin mutation. These mice did not have the altered lens phenotype, and served as a useful control. We speculated based on previous work by others [58-60] that the neo^r ^gene might exhibit diminished activity of mutant R49C gene that would provide novel insights into the biology of R49C αA-crystallin *in vivo*. To determine the effect of the R49C^neo ^gene on α-crystallin protein expression, lenses were analyzed by gel permeation chromatography. Figure 2 shows the chromatography profile of water-soluble lens proteins of WT/WT^neo ^and WT/R49C^neo ^mice measured by absorbance measurements at 280 nm. The analysis showed a 30% decrease in expression of total α-crystallin protein in WT/R49C^neo ^heterozygous lens as compared with wild type lenses. The expression of β-crystallins also decreased whereas γ-crystallin expression was not appreciably affected. As compared with WT/R49C lenses [42], α-crystallin decreased more in the WT/R49C^neo ^lenses.

**Figure 3:**
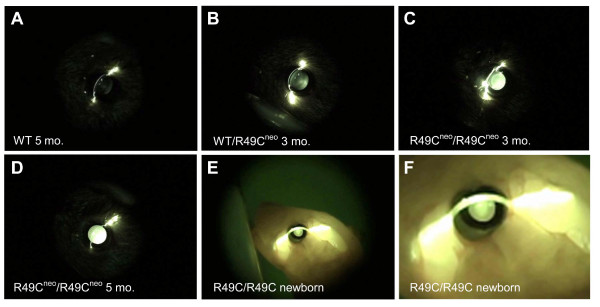
**Lens phenotypes in R49C^neo ^mice**. *(A-D) *Eyes were dilated and examined by slit lamp. *(A) *Wild type mice (5 months old) had clear lenses (stage 0). *(B) *Heterozygous WT/R49C^neo ^αA-crystallin knock-in mice (3 months old) had opacity in the posterior and nuclear regions of the lens (stage 2). *(C) *Homozygous R49C^neo^/R49C^neo ^αA-crystallin knock-in mice (3 months old) had a nearly complete cataract (stage 3–4). *(D) *R49C^neo^/R49C^neo ^αA-crystallin knock-in mouse lenses showed a complete cataract at 5 months (stage 4). *(E, F) *Cataract in lens of a newborn R49C/R49C homozygous mouse with deletion of the neo^r ^gene by Cre-recombinase. Slit lamp image *(E) *shows a severe nuclear opacity at birth (stage 3). *(F) *Higher magnification of the lens shown in *(E) *shows the nuclear opacity covering ~70% of the lens.

Lens opacities were confirmed in R49C^neo ^mutant mice by slit lamp analysis (Figure 3). By 3 months, R49C^neo ^mice showed evidence of opacities in posterior and nuclear regions. Over time, the cataract progressed to a nuclear cataract and then to an all-over opacity that included the cortical fibers (Figure 3A–D). Heterozygous mice at each age showed variable lens opacities ranging from clear (stage 0) at <2 months, stage 0 to stage 2 at 2–3 months, and clear to complete opacity (stage 4) at >4 months of age. Wild type mice did not show any of these abnormalities. At least four mice were examined for each genotype at a given age (Table 1A). The neomycin cassette was deleted by breeding homozygous R49C^neo ^mice with Cre-EIIa transgenic mice. These neomycin-deleted R49C mutant mice had a more severe lens phenotype, with homozygous R49C/R49C knock-in neo-deleted mice exhibiting an opacity that covered three-quarters of the young lens (Figure 3E and 3F). Overall, cataract progression was more rapid in R49C/R49C mice than in R49C^neo^/R49C^neo ^mice.

**Table 1:** Phenotypic changes in wild type (WT), WT/R49C^neo ^heterozygous, and R49C^neo^/R49C^neo ^homozygous mice.

A. Slit lamp analysis*					
Genotype	Phenotype	Age Group			Total
	
	Nuclear and posterior changes by slit lamp biomicroscopy	<2 month	2–3 months	>4 months	

WT		0/4	0/4	2/9	2/17 (0.12)
WT/R49C^neo^		3/6	14/22	7/7	24/35 (0.68)
R49C^neo^/R49C^neo^		4/5	5/7	13/14	22/26 (0.85)

B. Histological analysis*					

Genotype	Phenotype	Age Group			Total
	
	Swollen cells and/or posterior changes by histology	<1 month	2–3 months	>4 months	

WT		0/5	1/4	1/7	2/16 (0.13)
WT/R49C^neo^		4/11	2/4	2/3	8/18 (0.44)
R49C^neo^/R49C^neo^		4/5	3/4	6/8	13/17 (0.76)

* For each age group, the total number of mice analyzed is shown in the denominator, and the number of mice demonstrating the phenotype is shown in the numerator. The final columns show the total number of mice of each genotype that demonstrate a phenotype, with the proportion given in parentheses.

**Figure 4:**
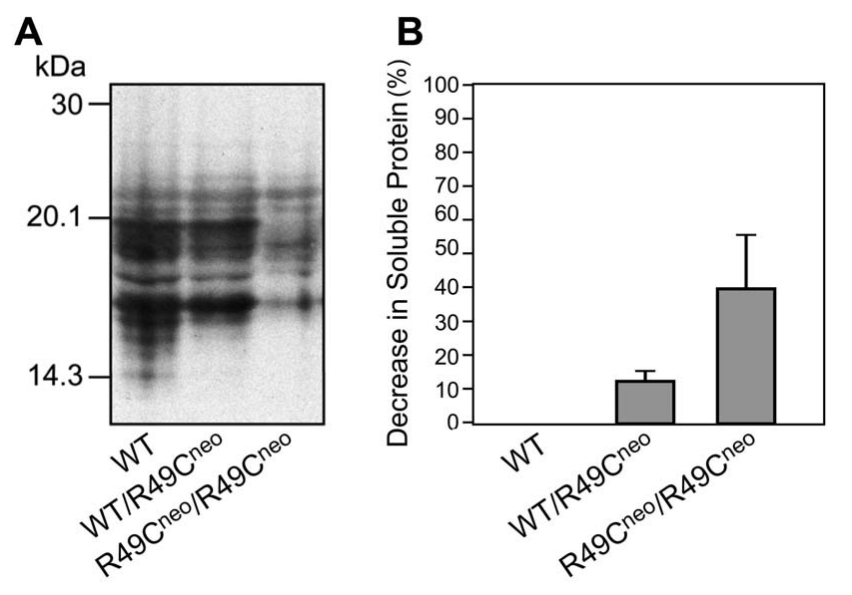
**Water-soluble αA-crystallin in R49C^neo ^knock-in lenses**. Protein solubility was assessed in lenses with different nuclear cataract severity ranging from stage 1 to 3 as determined by slit lamp analysis. *(A) *WT lenses had the highest protein solubility by immunoblotting with an antibody to α-crystallin. The wild type is clear lens (stage 0). A decrease in soluble protein levels was seen in the WT/R49C^neo ^lenses. R49C^neo^/R49C^neo ^lenses had a significantly lower water solubility. Proteins were overloaded so that even a small amount of protein could be visualized. Under these conditions, cross-reactivity of the antibody with other crystallins was observed. *(B) *The decrease in levels of soluble proteins was determined by densitometric scanning of immunoblots. The data represent an average of three independent experiments with approximately 3-month-old mouse lenses. Three pairs of lenses were analyzed for wild type and R49C^neo^/R49C^neo ^homozygous mice. Two pairs of lenses were analyzed for WT/R49C^neo ^heterozygous mice.

Examination of αA-crystallin soluble protein revealed that some of these changes were due to protein insolubility (Figure 4). Mice with nuclear opacity ranging from stage 1 to stage 3 (by slit lamp analysis) were separated into water-soluble and insoluble fractions. Water-soluble fractions were analyzed by immunoblotting. An excess of protein extract was loaded so that protein would be detectable even in the homozygous lenses. Protein insolubility was proportional to nuclear cataract formation by slit lamp analysis.

**Figure 5:**
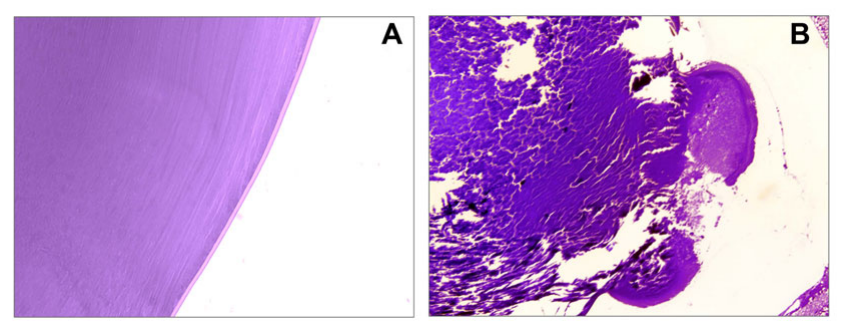
**Posterior lens defects in R49C^neo ^knock-in mouse lenses**. Lens sections were stained with toluidine blue and examined by bright field microscopy. *(A) *Lens posterior in 3-month-old wild type mouse appeared normal. *(B) *Lens posterior of a 3-month-old R49C^neo^/R49C^neo ^homozygous mouse showed a ruptured posterior capsule.

**Figure 6:**
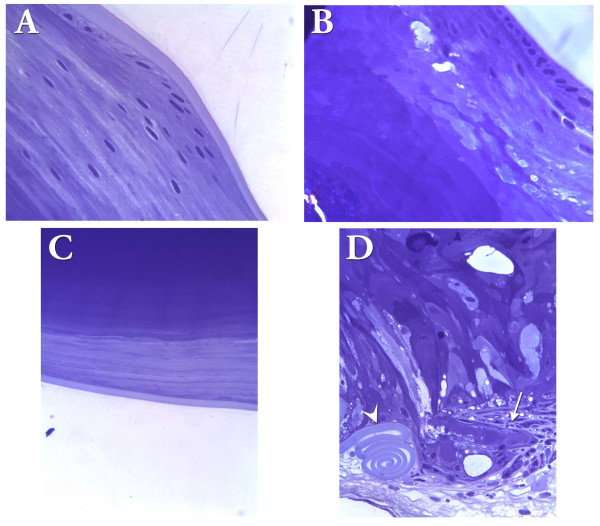
**Posterior lens defects in R49C^neo ^knock-in mouse**. Images are shown for lens sections from 9.5-month-old WT/WT^neo ^and WT/R49C^neo ^mouse. *(A) *Brightfield image of the equatorial region in a WT/WT^neo ^mid-sagittal lens section. *(B) *Brightfield image of the equatorial region in a WT/R49C^neo ^mid-sagittal lens section. *(C) *Posterior region of a WT/WT^neo ^lens shows a normal posterior capsule. *(D) *A WT/R49C^neo^lens with posterior cataract. Note the extensive posterior rupture, migration of cells and cell debris *(arrow)*, and compaction of the ruptured posterior capsule *(arrowhead)*.

Examples of the posterior lens changes observed in R49C^neo ^αA-crystallin knock-in lens sections are shown in Figures 5 and 6. Posterior cataract was evident in 3-month-old R49C^neo^/R49C^neo ^homozygous mice. At 9 months, WT/R49C^neo ^heterozygous mouse lenses showed severe posterior rupture, curling up of the posterior capsule, and migration of cells to the posterior lens (Figure 6).

**Figure 7:**
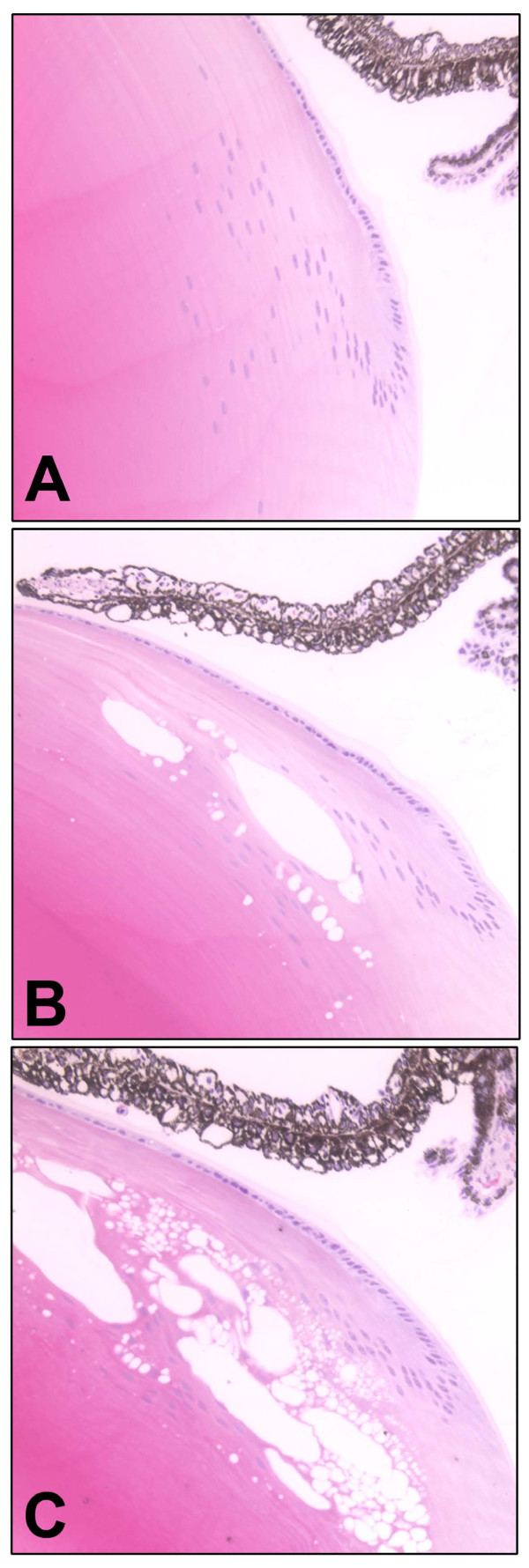
**Morphological alterations in cortical fiber cell membranes of R49C^neo ^lenses**. Lens sections were stained with haematoxylin/eosin. *(A) *Normal appearance of fiber cells in the lens equatorial region in 3-week-old wild type lenses. *(B) *Swollen fiber cells and separations between fiber cells in 3-week-old WT/R49C^neo ^heterozygous lenses. *(C) *Gaps between fiber cells in 3-week-old R49C^neo^/R49C^neo ^homozygous lenses.

At 3 weeks, histological examination of lenses revealed the presence of both small and large swollen cells or vacuoles in the lens cortical fibers of WT/R49C^neo ^mice, which were even more evident in R49C^neo ^homozygous mouse lenses (Figure 7). Early fiber formation appeared to occur normally. Swollen fiber cells begin to appear at 3 weeks postnatal in WT/R49C^neo ^heterozygous mice. Swollen fiber cells were confined to a distinct band of cortical fibers ~100 μm from the lens surface in both young and old mice. Fiber cells on either side of the swollen fibers appear to be unaffected. These swollen cells appeared to be formed by membrane rupture and fusion of cytoplasmic contents from multiple cells. Extensive undulations and interdigitations of these membranes as well as large separations between fiber cells occurred in homozygous mouse lenses. These gaps are evident in fiber cells that have detached from the capsule (Figure 6C). The proportion of swollen cells and posterior changes in WT/R49C^neo ^and R49C^neo^/R49C^neo ^mice examined by histology is shown in Table 1B.

**Figure 8:**
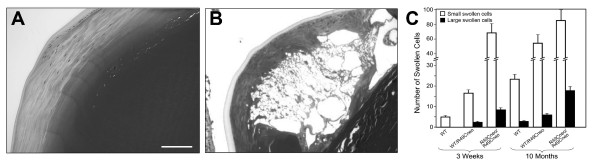
**Morphological alterations in lenses from older wild type and R49C^neo^/R49C^neo ^knock-in mice**. *(A) *Lens from wild type 10-month-old mouse with normal fiber cells near the lens surface and normal appearing fibers. *(B) *Large swollen cells and gaps between fiber cells in lens from 10-month-old R49C^neo^/R49C^neo ^mouse. *(C) *The swollen fiber cells increased in both size and number with increased age, and extended towards the center of the lens. The number of swollen cells per lens section is shown.

The number of swollen fiber cells increased in lenses of older homozygous mice, and extended towards the center of the lens (Figure 8). At 10 months old, a large area of aberrant fiber cells was observed. Fiber cells near the lens surface appeared normal. Newly synthesized cortical fibers appeared to form normally, but vacuoles were apparent in a band of fibers in the deep cortex 100 μm from lens surface, and increased dramatically towards the lens center.

**Figure 9:**
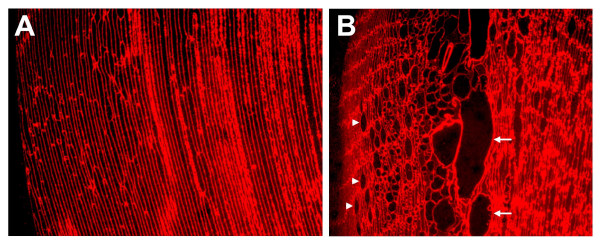
**Immunofluorescence analysis of fiber cells in wild type and R49C^neo^/R49C^neo ^knock-in lenses**. Immunofluorescence staining of lens sections with an antibody to membrane intrinsic protein MIP and an Alexa^568^-conjugated secondary antibody *(red) *revealed a normal fiber cell morphology in wild type lenses *(A)*, whereas the R49C^neo^/R49C^neo ^lenses *(B) *showed disorganized fiber cells and swollen cells *(arrows) *enclosed by membranes. Nuclei are shown by *arrowheads*.

**Figure 10:**
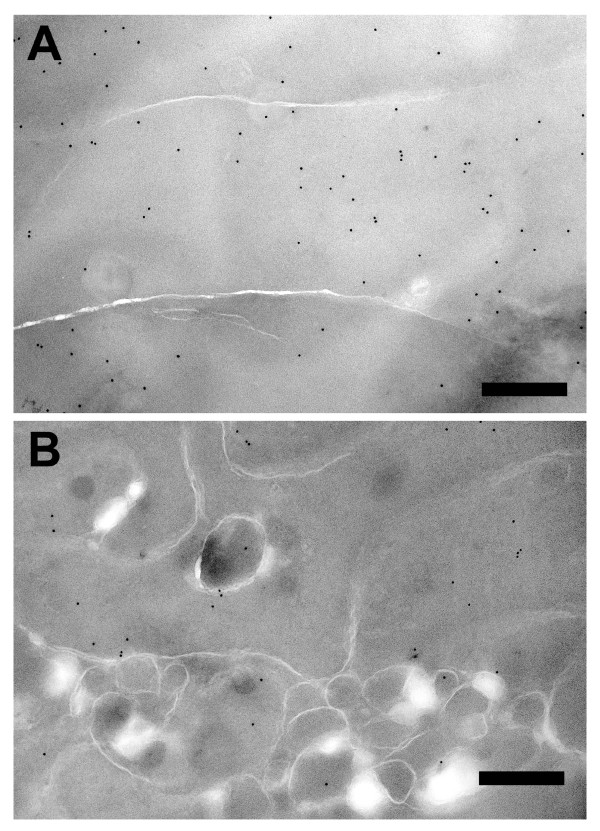
**Cryoimmuno electron microscopic analysis of wild type and R49C^neo^/R49C^neo ^knock-in lenses**. Lenses expressing either wild type or heterozygous R49C^neo^/R49C^neo ^αA-crystallin were labeled with an antibody to αA-crystallin and analyzed by cryoimmuno electron microscopy. *(A) *Wild type lenses showed linear fiber cell membranes. Immunogold labeling (black dots) showed that αA-crystallin was restricted to the cytoplasm. *(B) *Heterozygous WT/R49C^neo ^αA-crystallin lenses had several gold particles on the fiber cell membranes. Furthermore, the linearity of lens fiber cell membranes was lost, and significant gaps between cells were apparent.

Immunofluorescence analysis with an antibody to membrane intrinsic protein (MIP) revealed that the swollen cells are enclosed by fiber cell membranes (Figure 9). Swollen cells were not found in wild type littermates or in WT^neo ^mice. Opacification was related to defects in membrane structure of cortical lens fiber cells. Lenses were further examined by cryoimmuno electron microscopy with an antibody to αA-crystallin. Wild type lenses showed smooth and linear plasma membranes between the fiber cells, and αA-crystallin was restricted to the cytoplasm (Figure 10A). In contrast, fiber cell membranes of heterozygous WT/R49C^neo ^lenses were non-linear with extensive undulations, and significant gaps between the cells. A greater proportion of the αA-crystallin immunoreactivity was associated with fiber cell membranes in the heterozygous lenses (Figure 10B) than in wild type lenses.

## Discussion

In human patients with a missense mutation in exon 1 of the gene encoding αA-crystallin *(CRYAA)*, autosomal dominant nuclear cataract was shown to segregate in a four generation Caucasian family [1]. The C to T transition in the first base of codon 49 of *CRYAA *results in the non-conservative substitution of arginine 49 to cysteine (R49C) in αA-crystallin protein [1]. Patients with cataracts are heterozygous for this mutation, which suggests that both wild type and R49C mutant αA-crystallin subunits are expressed in these lenses in addition to wild type αB-crystallin. Unfortunately, photographic documentation of the cataracts is not available, preventing a detailed phenotypic analysis. It was shown previously that the αA-crystallin R49C mutation causes lens opacities in a knock-in mouse model, due to protein insolubilization and cell death [42,55]. Here a R49C^neo ^construct was used that causes a milder lens phenotype to show that R49C αA-crystallin disrupts normal fiber cell organization and structure.

Homozygous mice with deletion of the floxed neo^r ^gene (R49C/R49C) have a more drastic phenotype, with smaller eyes and smaller lenses than wild type and heterozygous (WT/R49C) mutant mice [55]. This implies that the neo^r ^gene is suppressing expression of the R49C-αA mutant protein, and is supported by FPLC analysis of soluble lens proteins. Indeed, previous studies indicate that selectable markers inserted in non-coding regions can affect gene expression at both the DNA and RNA level, resulting in reduced protein expression [58-60].

The lenses of WT/R49C^neo ^and R49C^neo^/R49C^neo ^mice displayed remarkable posterior lens defects and accumulation of swollen cells in the lens cortex, and developed nuclear cataracts. Both posterior lens cell defects and number of swollen fiber cells increased with age and with R49C^neo ^dosage, with more defects in R49C^neo^/R49C^neo ^mice than in WT/R49C^neo ^mice. The appearance of swollen fiber cells in the lens cortex may be an early marker of structural perturbation in the R49C^neo ^lenses. The swollen fiber cells in deep cortical fibers of R49C^neo ^lenses were evident as early as 3 weeks postnatal and increased with age, such that more than half of the lens was covered with swollen fiber cells at 10 months.

Histological and immunofluorescence analysis of the R49C^neo ^knock-in lenses suggests that the lens opacities observed in R49C^neo ^mice are associated with membrane defects in the deep cortical fiber cells approximately 100 μm from the lens surface that result in gaps between adjacent fiber cells. Visible swelling or vacuolar areas are often limited to one or more segments of a fiber cell; hence it is likely that there are many more aberrant fiber cells than those observed in a single mid-sagittal section. Vacuolated cells indicate destruction of the fiber cells. Further studies are necessary to determine whether these vacuolar or swollen cells are formed by membrane rupture and fusion of cytoplasmic contents from multiple cells. This type of fiber cell swelling has been reported in diabetic and other models of cataract [61,62]. Membrane defects have also been shown to be associated with lens opacities induced by dexamethasone and mechanical stress in cultured lenses, both of which are associated with the loss of cadherin junctions [17,63]. The interaction between αA-crystallin and fiber cell membranes is well-established [16,64,65], and has been shown to increase with lens aging and in cataracts [66]. In the current study, membrane alterations in WT/R49C^neo ^mouse lenses were confirmed by ultrastructural analysis. Cryoimmuno electron microscopic analysis of heterozygous R49C^neo ^lenses with an antibody specific to αA-crystallin showed loss of membrane linearity in the cortical fiber cells, undulations of the membranes, and significant gaps between cells. Furthermore, more αA-crystallin was membrane-associated in the heterozygous knock-in lens fiber cells than in the wild type controls. Our studies also showed that the solubility of αA-crystallin is decreased in the R49C^neo ^lenses, which may have a profound effect on its binding to lens fiber cell membranes [67].

Evidence in the literature suggests that loss of α-crystallin function through gene knockout or mutation alters protein homeostasis by increasing the abundance of aggregation-prone proteins. This may result in altered protein-protein interactions, and protein insolubility [55,68]. Correct protein conformation is essential for normal cell function, and proteotoxic stress due to protein aggregation and loss of protein homeostasis is known to be associated with aging and disease [69].

In the present work, no significant defects were found in WT/WT^neo ^and WT^neo^/WT^neo ^lenses. In targeted gene deletion studies in mice, αA+/- heterozygous mouse lenses do not show lens opacification, whereas αA-/- homozygous lenses develop cataracts at an early age [24]. Most of the defects in αA-crystallin knockout (αA-/-) mouse lenses are due to protein insolubility in the lens nucleus and cell death in the lens epithelium [24,31]; no defects in fiber cell elongation or fiber cell morphology were reported. Our previous study showed that lens defects in R49C/R49C knock-in mice are the result of a drastic alteration of lens fiber cell morphology, although early fiber cell elongation is normal [55]. In the present study, we did not observe as strong an effect on lens fiber morphology in young R49C^neo ^mice as in R49C/R49C mice, but did observe varied posterior lens defects beginning at an early postnatal age, as well as posterior migration of nuclei at older ages and posterior rupture. Such posterior migration of epithelial cells has been reported in human posterior subcapsular cataracts [70,71]. A disorganization of the postequatorial fiber zone was also observed, which has been reported in human posterior subcapsular cataracts. Thus, the R49C^neo ^mouse model system appears to be an effective model for human posterior subcapsular cataracts.

## Conclusion

In summary, the results of the present work, combined with previously published data, provide evidence that αA-crystallin plays a role in the structure of fiber cell membranes, and is involved in the formation of hereditary cataracts. The present study provides insight into the *in vivo *effects of the R49C mutation of αA-crystallin on the lens, and extends the findings of previous studies [1,42]. In mice, expression of the R49C αA-crystallin mutant protein in the presence of the neo^r ^gene causes posterior cataracts with posterior migration of epithelial cell nuclei, and demonstrates that the mutant protein affects the integrity of lens fiber cells in the deep cortex. These changes are milder than those observed when the neo^r ^gene is deleted. By studying homozygous R49C^neo^/R49C^neo ^mice that begin to lose transparency around 2 months of age, compared with the typical onset at birth in R49C/R49C mice, the author has obtained additional information about the progression of cataracts. The R49C^neo ^mice described in the present study are one of only a few models available to study hereditary posterior cataract, a common human lens pathology.

## Competing interests

The author declares no competing interests.

## Authors' contributions

The author performed slit lamp biomicroscopy, microscopic analysis of lens sections, and drafted the manuscript and subsequent revisions

## Pre-publication history

The pre-publication history for this paper can be accessed here:



## References

[B1] Mackay DS, Andley UP, Shiels A (2003). Cell death triggered by a novel mutation in the alphaA-crystallin gene underlies autosomal dominant cataract linked to chromosome 21q. Eur J Hum Genet.

[B2] Graw J (2004). Congenital hereditary cataracts. Int J Dev Biol.

[B3] Graw J (2009). Genetics of crystallins: cataract and beyond. Exp Eye Res.

[B4] Hejtmancik JF, Smaoui N (2003). Molecular genetics of cataract. Dev Ophthalmol.

[B5] Shiels A, Hejtmancik JF (2007). Genetic origins of cataract. Arch Ophthalmol.

[B6] Bloemendal H, de Jong W, Jaenicke R, Lubsen NH, Slingsby C, Tardieu A (2004). Ageing and vision: structure, stability and function of lens crystallins. Prog Biophys Mol Biol.

[B7] Ingolia TD, Craig EA (1982). Four small Drosophila heat shock proteins are related to each other and to mammalian alpha-crystallin. Proc Natl Acad Sci USA.

[B8] Horwitz J (1992). Alpha-crystallin can function as a molecular chaperone. Proc Natl Acad Sci USA.

[B9] Horwitz J (2009). Alpha crystallin: The quest for a homogeneous quaternary structure. Exp Eye Res.

[B10] Aquilina JA, Benesch JL, Bateman OA, Slingsby C, Robinson CV (2003). Polydispersity of a mammalian chaperone: mass spectrometry reveals the population of oligomers in alphaB-crystallin. Proc Natl Acad Sci USA.

[B11] Ghosh JG, Shenoy AK, Clark JI (2006). N- and C-Terminal motifs in human alphaB crystallin play an important role in the recognition, selection, and solubilization of substrates. Biochemistry.

[B12] Sun TX, Akhtar NJ, Liang JJ (1998). Subunit exchange of lens alpha-crystallin: a fluorescence energy transfer study with the fluorescent labeled alphaA-crystallin mutant W9F as a probe. FEBS Lett.

[B13] Sun TX, Liang JJ (1998). Intermolecular exchange and stabilization of recombinant human alphaA- and alphaB-crystallin. J Biol Chem.

[B14] Van Montfort R, Slingsby C, Vierling E (2001). Structure and function of the small heat shock protein/alpha-crystallin family of molecular chaperones. Adv Protein Chem.

[B15] Cobb BA, Petrash JM (2002). alpha-Crystallin chaperone-like activity and membrane binding in age-related cataracts. Biochemistry.

[B16] Ifeanyi F, Takemoto L (1990). Alpha crystallin from human cataractous vs. normal lenses: change in binding to lens membrane. Exp Eye Res.

[B17] Zhou J, Leonard M, Van Bockstaele E, Menko AS (2007). Mechanism of Src kinase induction of cortical cataract following exposure to stress: destabilization of cell-cell junctions. Mol Vis.

[B18] MacRae TH (2000). Structure and function of small heat shock/alpha-crystallin proteins: established concepts and emerging ideas. Cell Mol Life Sci.

[B19] Maddala R, Rao VP (2005). alpha-Crystallin localizes to the leading edges of migrating lens epithelial cells. Exp Cell Res.

[B20] Perng MD, Wen SF, van den IP, Prescott AR, Quinlan RA (2004). Desmin aggregate formation by R120G alphaB-crystallin is caused by altered filament interactions and is dependent upon network status in cells. Mol Biol Cell.

[B21] Quinlan R (2002). Cytoskeletal competence requires protein chaperones. Prog Mol Subcell Biol.

[B22] Xi JH, Bai F, McGaha R, Andley UP (2006). Alpha-crystallin expression affects microtubule assembly and prevents their aggregation. Faseb J.

[B23] Robinson ML, Overbeek PA (1996). Differential expression of alpha A- and alpha B-crystallin during murine ocular development. Invest Ophthalmol Vis Sci.

[B24] Brady JP, Garland D, Duglas-Tabor Y, Robison WG, Groome A, Wawrousek EF (1997). Targeted disruption of the mouse alpha A-crystallin gene induces cataract and cytoplasmic inclusion bodies containing the small heat shock protein alpha B-crystallin. Proc Natl Acad Sci USA.

[B25] Brady JP, Garland DL, Green DE, Tamm ER, Giblin FJ, Wawrousek EF (2001). AlphaB-crystallin in lens development and muscle integrity: a gene knockout approach. Invest Ophthalmol Vis Sci.

[B26] Andley UP (2007). Crystallins in the eye: Function and pathology. Prog Retin Eye Res.

[B27] Andley UP, Song Z, Wawrousek EF, Bassnett S (1998). The molecular chaperone alphaA-crystallin enhances lens epithelial cell growth and resistance to UVA stress. J Biol Chem.

[B28] Srinivasan AN, Nagineni CN, Bhat SP (1992). alpha A-crystallin is expressed in non-ocular tissues. J Biol Chem.

[B29] Andley UP, Song Z, Wawrousek EF, Fleming TP, Bassnett S (2000). Differential protective activity of alpha A- and alphaB-crystallin in lens epithelial cells. J Biol Chem.

[B30] Morozov V, Wawrousek EF (2006). Caspase-dependent secondary lens fiber cell disintegration in alphaA-/alphaB-crystallin double-knockout mice. Development.

[B31] Xi JH, Bai F, Andley UP (2003). Reduced survival of lens epithelial cells in the alphaA-crystallin-knockout mouse. J Cell Sci.

[B32] Litt M, Kramer P, LaMorticella DM, Murphey W, Lovrien EW, Weleber RG (1998). Autosomal dominant congenital cataract associated with a missense mutation in the human alpha crystallin gene CRYAA. Hum Mol Genet.

[B33] Liu Y, Zhang X, Luo L, Wu M, Zeng R, Cheng G, Hu B, Liu B, Liang JJ, Shang F (2006). A Novel {alpha}B-Crystallin Mutation Associated with Autosomal Dominant Congenital Lamellar Cataract. Invest Ophthalmol Vis Sci.

[B34] Pras E, Frydman M, Levy-Nissenbaum E, Bakhan T, Raz J, Assia EI, Goldman B, Pras E (2000). A nonsense mutation (W9X) in CRYAA causes autosomal recessive cataract in an inbred Jewish Persian family. Invest Ophthalmol Vis Sci.

[B35] Vanita V, Singh JR, Hejtmancik JF, Nuernberg P, Hennies HC, Singh D, Sperling K (2006). A novel fan-shaped cataract-microcornea syndrome caused by a mutation of CRYAA in an Indian family. Mol Vis.

[B36] Vicart P, Caron A, Guicheney P, Li Z, Prevost MC, Faure A, Chateau D, Chapon F, Tome F, Dupret JM (1998). A missense mutation in the alphaB-crystallin chaperone gene causes a desmin-related myopathy. Nat Genet.

[B37] Richter L, Flodman P, Barria von-Bischhoffshausen F, Burch D, Brown S, Nguyen L, Turner J, Spence MA, Bateman JB (2008). Clinical variability of autosomal dominant cataract, microcornea and corneal opacity and novel mutation in the alpha A crystallin gene (CRYAA). Am J Med Genet A.

[B38] Andley UP (2006). Crystallins and hereditary cataracts: molecular mechanisms and potential for therapy. Expert Rev Mol Med.

[B39] Santhiya ST, Soker T, Klopp N, Illig T, Prakash MV, Selvaraj B, Gopinath PM, Graw J (2006). Identification of a novel, putative cataract-causing allele in CRYAA (G98R) in an Indian family. Mol Vis.

[B40] Koteiche HA, McHaourab HS (2006). Mechanism of a hereditary cataract phenotype. Mutations in alphaA-crystallin activate substrate binding. J Biol Chem.

[B41] Treweek TM, Rekas A, Lindner RA, Walker MJ, Aquilina JA, Robinson CV, Horwitz J, Perng MD, Quinlan RA, Carver JA (2005). R120G alphaB-crystallin promotes the unfolding of reduced alpha-lactalbumin and is inherently unstable. Febs J.

[B42] Andley UP, Hamilton PD, Ravi N (2008). Mechanism of insolubilization by a single-point mutation in alphaA-crystallin linked with hereditary human cataracts. Biochemistry.

[B43] Bova MP, Yaron O, Huang Q, Ding L, Haley DA, Stewart PL, Horwitz J (1999). Mutation R120G in alphaB-crystallin, which is linked to a desmin-related myopathy, results in an irregular structure and defective chaperone-like function. Proc Natl Acad Sci USA.

[B44] Murugesan R, Santhoshkumar P, Sharma KK (2007). Cataract-causing alphaAG98R mutant shows substrate-dependent chaperone activity. Mol Vis.

[B45] Shroff NP, Cherian-Shaw M, Bera S, Abraham EC (2000). Mutation of R116C results in highly oligomerized alpha A-crystallin with modified structure and defective chaperone-like function. Biochemistry.

[B46] Singh D, Raman B, Ramakrishna T, Rao Ch M (2007). Mixed oligomer formation between human alphaA-crystallin and its cataract-causing G98R mutant: structural, stability and functional differences. J Mol Biol.

[B47] de Jong WW, Caspers GJ, Leunissen JA (1998). Genealogy of the alpha-crystallin – small heat-shock protein superfamily. Int J Biol Macromol.

[B48] Biswas A, Miller A, Oya-Ito T, Santhoshkumar P, Bhat M, Nagaraj RH (2006). Effect of site-directed mutagenesis of methylglyoxal-modifiable arginine residues on the structure and chaperone function of human alphaA-crystallin. Biochemistry.

[B49] Hsu CD, Kymes S, Petrash JM (2006). A transgenic mouse model for human autosomal dominant cataract. Invest Ophthalmol Vis Sci.

[B50] Andley UP, Patel HC, Xi JH (2002). The R116C mutation in alpha A-crystallin diminishes its protective ability against stress-induced lens epithelial cell apoptosis. J Biol Chem.

[B51] Graw J, Loster J, Soewarto D, Fuchs H, Meyer B, Reis A, Wolf E, Balling R, Hrabe de Angelis M (2001). Characterization of a new, dominant V124E mutation in the mouse alphaA-crystallin-encoding gene. Invest Ophthalmol Vis Sci.

[B52] Graw J (1999). Cataract mutations and lens development. Prog Retin Eye Res.

[B53] Graw J (2003). The genetic and molecular basis of genetic eye defects. Nature Reviews Genetics.

[B54] Xia CH, Liu H, Chang B, Cheng C, Cheung D, Wang M, Huang Q, Horwitz J, Gong X (2006). Arginine 54 and Tyrosine 118 residues of {alpha}A-crystallin are crucial for lens formation and transparency. Invest Ophthalmol Vis Sci.

[B55] Xi JH, Bai F, Gross J, Townsend RR, Menko AS, Andley UP (2008). Mechanism of small heat shock protein function in vivo: a knock-in mouse model demonstrates that the R49C mutation in alpha A-crystallin enhances protein insolubility and cell death. J Biol Chem.

[B56] Andley UP, Mathur S, Griest TA, Petrash JM (1996). Cloning, expression, and chaperone-like activity of human alphaA-crystallin. J Biol Chem.

[B57] Bai F, Xi JH, Andley UP (2007). Up-regulation of tau, a brain microtubule-associated protein, in lens cortical fractions of aged alphaA-, alphaB-, and alphaA/B-crystallin knockout mice. Mol Vis.

[B58] Meyers EN, Lewandoski M, Martin GR (1998). An Fgf8 mutant allelic series generated by Cre- and Flp-mediated recombination. Nat Genet.

[B59] Scacheri PC, Crabtree JS, Novotny EA, Garrett-Beal L, Chen A, Edgemon KA, Marx SJ, Spiegel AM, Chandrasekharappa SC, Collins FS (2001). Bidirectional transcriptional activity of PGK-neomycin and unexpected embryonic lethality in heterozygote chimeric knockout mice. Genesis.

[B60] Shannon MB, Patton BL, Harvey SJ, Miner JH (2006). A hypomorphic mutation in the mouse laminin alpha5 gene causes polycystic kidney disease. J Am Soc Nephrol.

[B61] Bond J, Green C, Donaldson P, Kistler J (1996). Liquefaction of cortical tissue in diabetic and galactosemic rat lenses defined by confocal laser scanning microscopy. Invest Ophthalmol Vis Sci.

[B62] Tunstall MJ, Eckert R, Donaldson P, Kistler J (1999). Localised fibre cell swelling characteristic of diabetic cataract can be induced in normal rat lens using the chloride channel blocker 5-nitro-2-(3-phenylpropylamino) benzoic acid. Ophthalmic Res.

[B63] Lyu J, Kim JA, Chung SK, Kim KS, Joo CK (2003). Alteration of cadherin in dexamethasone-induced cataract organ-cultured rat lens. Invest Ophthalmol Vis Sci.

[B64] Ifeanyi F, Takemoto L (1990). Specificity of alpha crystallin binding to the lens membrane. Curr Eye Res.

[B65] Ifeanyi F, Takemoto L (1991). Involvement of the N-terminal region in alpha-crystallin-lens membrane recognition. Exp Eye Res.

[B66] Boyle DL, Takemoto L (1996). EM immunolocalization of alpha-crystallins: association with the plasma membrane from normal and cataractous human lenses. Curr Eye Res.

[B67] Cobb BA, Petrash JM (2000). Structural and functional changes in the alpha A-crystallin R116C mutant in hereditary cataracts. Biochemistry.

[B68] McHaourab HS, Dodson EK, Koteiche HA (2002). Mechanism of chaperone function in small heat shock proteins. Two-mode binding of the excited states of T4 lysozyme mutants by alphaA-crystallin. J Biol Chem.

[B69] Morimoto RI (2008). Proteotoxic stress and inducible chaperone networks in neurodegenerative disease and aging. Genes Dev.

[B70] Eshaghian J, Streeten BW (1980). Human posterior subcapsular cataract. An ultrastructural study of the posteriorly migrating cells. Arch Ophthalmol.

[B71] Streeten BW, Eshaghian J (1978). Human posterior subcapsular cataract. A gross and flat preparation study. Arch Ophthalmol.

